# Alterations in Metabolites Associated With Umbilical Cord Blood in Monozygotic Twins Discordant for Congenital Heart Disease

**DOI:** 10.1002/pdi3.70042

**Published:** 2026-03-13

**Authors:** Fang Xiang, Xiao Yuan, Yi Yang, Philip N. Baker, Mark D. Kilby, Chao Tong, Xi Yuan

**Affiliations:** ^1^ State Key Laboratory of Maternal and Fetal Medicine of Chongqing Municipality Chongqing Medical University Chongqing China; ^2^ Growth, Development and Mental Health Center of Children and Adolescents, National Clinical Research Center for Child Health and Disorders, Ministry of Education Key Laboratory of Child Development and Disorders Children's Hospital of Chongqing Medical University Chongqing China; ^3^ Faculty of Medicine and Health Sciences University of East Anglia Norwich Research Park Norwich UK; ^4^ Fetal Medicine Centre Birmingham Women's & Children's Foundation Trust Birmingham UK; ^5^ College of Medical & Health Sciences University of Birmingham Birmingham UK; ^6^ Early Life Developmental Programming Laboratory Children's Hospital of Chongqing Medical University Chongqing China

**Keywords:** congenital heart diseases, metabolomics, twin study, umbilical cord blood

## Abstract

The specific mechanisms and screening methods for congenital heart disease (CHD) remain elusive. Evidence indicates that aberrant maternal metabolomic profiles are associated with CHD, but it is uncertain whether such an association exists in umbilical cord blood. This study aimed to measure the metabolome shifts in monozygotic (MZ) twins discordant for CHD and, if present, identify the altered metabolites and metabolic pathways. Umbilical cord blood from three pairs of MZ twins discordant for CHD, identified through the prospective, population‐based longitudinal twin study (LoTiS), was subjected to ultrahigh‐performance liquid chromatography coupled with mass spectrometry (UHPLC‐MS/MS) based metabolomic analysis. Orthogonal partial least square‐discriminate analysis (OPLS‐DA) and random forest (RF) analysis were used to determine differences in metabolic profiles and individual metabolites. In univariate analysis, two specific metabolites were identified in the umbilical cord blood of CHD cases compared to their MZ twins without complications. After the paired‐sample *t*‐test, four differentially expressed metabolites (DEMs) were identified, three of which were closely related to fatty acids and their metabolism. Enrichment pathway analysis revealed dysregulation of various metabolic pathways, including glucose metabolism, lipid metabolism, and amino acid metabolism pathways, in MZ twins with CHD compared to their healthy counterparts. The results demonstrated that the metabolomic signature of umbilical cord blood in CHD differs from that of their healthy MZ co‐twins, and the revelation of associated metabolites and pathways provide preliminary clues for generating hypotheses about the metabolic correlates of CHD predisposition. Findings are limited by small sample size and require validation in larger, independent cohorts.

## Graphical abstract

This study found that the metabolomic signature of umbilical venous cord blood in congenital heart disease (CHD) differs from that of healthy monozygotic (MZ) co‐twins. Dysregulation of metabolic pathways like glucose, lipid, and amino acid metabolism, along with altered metabolites, helps understand CHD predisposition. These metabolite differences, identified in the cord blood of 3 discordant MZ twin pairs, are illustrated to link the in utero metabolic environment to the development of CHD.

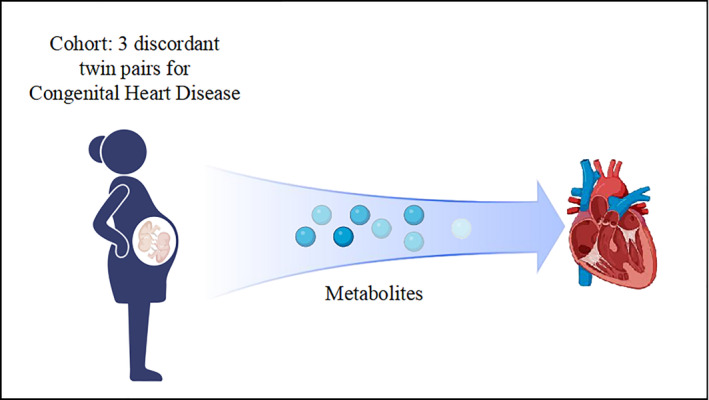

## 1 Introduction

Globally, congenital anomalies affect approximately 2% of total births, with congenital heart disease (CHD) as the most common anomaly. The overall estimated prevalence of CHD is 1%, with constant rates across the world [1, 2]. CHD is associated with significant infant morbidity and mortality [3]. Although gene alterations have been associated with fetal cardiac malformations [4, 5], little is known about how nongenetic contributors, such as the metabolome, impact cardiac development. The integrative capacity of metabolomics has the potential to uncover unifying and potentially targetable metabolic phenotypes generated by a broad diversity of upstream systemic inputs [6].

Metabolomics is the systematic study of metabolites—small molecules, including substrates, intermediates, and products of cell metabolism. It aims to provide a comprehensive “metabolic profile” of a biological system at a given time, reflecting its physiological state and response to a disease. The high‐throughput measurement of these small metabolites and the use of informatics techniques to define them enable the characterization of metabolomic signatures [7, 8]. Metabolomics has provided unique insights into the molecular mechanisms underlying the pathogenesis of CHD. The amino acid metabolic pathway—including arginine metabolism, cysteine metabolism, and branched‐chain amino acid metabolism—has been described as significantly changed and different among adults with CHD [6, 9]. Metabolomic analysis in serum of patients with CHD has demonstrated that uric acid and sphingolipid were significantly increased, indicating not only oxidative stress and nitric oxide pathway alterations, but also subtle “markers” of myocardial dysfunction [9, 10, 11]. Additionally, most studies have focused on the alterations of metabolites in maternal peripheral blood of pregnancies complicated with CHD, and there is very little information on profiles within cord blood. The umbilical cord blood metabolome may offer information on the intrauterine environment involving the complex interaction of maternal and fetal metabolism, along with the effects of placental nutrient transfer [12, 13]. The Developmental Origin of Health and Disease (DOHaD) hypothesis states that later life diseases may be influenced by experiences and conditions during prenatal life in the in utero environment [14]. Exposure to detrimental environmental factors may induce (directly or indirectly) adverse health outcomes [15]. Accordingly, metabolomics analyses of cord blood may better assess the prenatal environment and the metabolic status of neonates.

Monozygotic (MZ) twins discordant for disease provide a natural case–control model for studying metabolomic associations while effectively controlling for confounding factors, such as germ line genetics, age, sex, and early life experiences [16]. Thus, in the present study, we employed a co‐twin control study design using ultrahigh‐performance liquid chromatography coupled with mass spectrometry (UHPLC‐MS/MS) to identify a global metabolomic signature of CHD in cord blood and further identify metabolites significantly associated with metabolic health. Notably, a key limitation of this study design is that CHD‐discordant MZ twins are rare at the population level, resulting in a small cohort sample size, which defines the exploratory nature of this investigation. Our goal was to explore whether this nontargeted profiling approach could identify individual compounds or their combinations as preliminary candidate markers linked to CHD, relative to the matched healthy co‐twins.

## 2 Methods

### 2.1 Patient Recruitment

The sample was derived from the population‐based Longitudinal Twin Study (LoTiS, trial registration number: ChiCTR‐OOC‐16008203). Women with naturally conceived twin pregnancies were recruited at 11–16 weeks of gestation, and their twins were enrolled in pediatric follow‐up after birth (MZ or dizygotic twins were confirmed by zygosity testing). The study protocol has been previously published [17]. In total, 439 twin pregnant women were recruited at the first follow‐up visit. Among them, there were 3 cases of MZ twins discordant for CHD. CHD was diagnosed based on ultrasound results interpreted in accordance with published guidelines; the diagnosis was performed between 18 and 24 weeks of gestational age by two senior pediatric cardiologists with over 10 years of experience in CHD diagnosis [18]. Exclusion criteria were as follows: (1) other major congenital malformations; (2) severe perinatal asphyxia (5‐min Apgar score < 4), severe infection, or neonatal metabolic diseases; (3) hemolyzed, contaminated, or insufficient (< 2 mL) samples; and (4) refusal/withdrawal of consent. Detailed clinical information of the twins with different CHD subtypes has been presented in earlier published work [19]. The study was conducted in accordance with the principles of the Declaration of Helsinki and was approved by the ethics committee of the First Affiliated Hospital of Chongqing Medical University. Written informed consent was obtained from those twins' mothers.

For sample processing, trained nurses collected umbilical cord blood from the umbilical vein into 10 mL EDTA anticoagulant tubes within a few minutes after delivery in a sterile environment. Blood specimens were promptly transported to lab on ice and processed within 1 h to preserve metabolomic stability. Upon arrival, blood samples were centrifuged at 3000 × g for 15 min at 4°C to separate plasma, which was promptly collected and aliquoted into sterile, enzyme‐free cryopreservation tubes. The plasma was snap‐frozen and stored at −80°C, with strict avoidance of repeated freeze–thaw. These stored plasma samples were subsequently sent to a specialized company for metabolomic analysis.

### 2.2 Metabolite Extraction From Cord Plasma

The collected plasma samples were thawed on ice and vortexed, and 100 μL of plasma was transferred into a new sterile polypropylene tube for use. To extract metabolites, each sample was vortex‐mixed with 400 μL prechilled methanol. The samples were incubated on ice for 5 min and then centrifuged at 15,000 rpm 4°C for 5 min. A portion of the supernatant was diluted to a final concentration containing 60% methanol with liquid chromatography‐mass spectrometry (LC‐MS) grade water. The samples were subsequently transferred to a fresh Eppendorf tube fitted with 0.22 μm filters and then centrifuged at 15,000 rpm, 4°C for 10 min. Finally, the filtrate was injected into the ultrahigh‐performance liquid chromatography‐tandem mass spectrometry (UHPLC‐MS/MS) system for analysis.

### 2.3 UHPLC‐MS/MS Analysis

UHPLC‐MS/MS analyses were performed using a Vanquish UHPLC system (Thermo Fisher) coupled with an Orbitrap *Q* Exactive HF‐X mass spectrometer (Thermo Fisher). Prior to injection, samples were thawed on ice and vortexed for 30 s to ensure homogeneity. A 5‐μL aliquot of each sample was injected onto a Hyperil Gold column (100 × 2.1 mm, 1.9 μm) maintained at 30°C, with a constant flow rate of 0.2 mL/min and a 16‐min linear gradient elution program. The eluent systems were optimized for different ion modes: For positive polarity mode, eluent A was 0.1% formic acid [FA] in water and eluent B was methanol; for negative polarity mode, eluent A was 5 mmol/L ammonium acetate (pH 9.0, adjusted with ammonia solution) and eluent B was methanol. The solvent gradient was set as follows: 2% B (0–1.5 min), linear increase from 2% to 100% B (1.5–13.5 min), hold at 100% B (13.5–15.5 min), linear decrease from 100% to 2% B (15.5–15.6 min), and hold at 2% B (15.6–16.0 min) for column re‐equilibration. The Orbitrap *Q* Exactive HF‐X mass spectrometer was operated in both positive and negative polarity modes with the following parameters: spray voltage of 3.2 kV (positive mode) and 2.8 kV (negative mode), capillary temperature of 320°C, sheath gas flow rate of 35 arbitrary units (a.u.), and auxiliary gas flow rate of 10 a.u. Mass spectral data were acquired in full scan mode (m/z range: 70–1050) to ensure comprehensive metabolite coverage.

### 2.4 Data Processing and Metabolite Identification

The raw data files generated by UHPLC‐MS/MS were processed using Compound Discoverer 3.0 software (CD3.0, Thermo Fisher Scientific) to perform peak alignment, peak picking, and quantitation for each metabolite. For metabolomic analysis, the main parameters were set as follows: retention time tolerance—0.2 min, absolute mass tolerance—5 ppm, signal intensity tolerance—30%, signal/noise (S/N) ratio—10, and minimum intensity threshold—100,000. After that, peak intensities were normalized to the total spectral intensity to minimize systematic variations. The normalized data were used to predict the molecular formula based on adduct ions, molecular ion peaks, and fragment ions. Peaks were then matched against the mzCloud (https://www.mzcloud.org/) and ChemSpider (http://www.chemspider.com/) databases to obtain accurate qualitative and relative quantitative results.

### 2.5 Quality Control of Metabolomics Analysis

As described previously [20], equal volumes of each plasma sample were pooled to prepare a quality control (QC) sample for metabolomic analysis, which was used to monitor instrument performance, stabilize the UHPLC‐MS/MS system, and evaluate system stability throughout the analytical process. The blank sample was designated for background subtraction to eliminate systematic noise from the LC‐MS/MS system. The QC sample was aliquoted into 200 μL portions, and these aliquots were interspersed among the experimental samples for UHPLC‐MS/MS analysis (i.e., before, during, and after the injection of experimental samples) to assess batch effects. A total of 11 reproducible metabolomic profiles were acquired from the QC aliquots. Pearson correlation coefficients among QC sample aliquots were calculated based on the relative quantification of metabolites. We calculated the coefficient of variation (CV) values of the metabolites in the QC sample, and metabolites with CV less than 30% were considered as the final identification results. Before statistical data analysis, a sample normalization was performed as previously described [21], using the following formula: normalized metabolite quantification = original metabolite quantification/(sum of all metabolite quantifications in the sample/sum of all metabolite quantifications in QC1). QC1 (quality control 1) refers to the first pooled quality control sample.

### 2.6 Data Analysis

The identified metabolites were annotated using the Kyoto Encyclopedia of Genes and Genomes (KEGG, https://www.genome.jp/kegg/pathway.html), the Human Metabolome Database (HMDB, https://hmdb.ca/metabolites), and the LIPID MAPS database (http://www.lipidmaps.org/). The processed data were assessed by orthogonal partial least squares discriminant analysis (OPLS‐DA) to identify differences between the CHD and NC (normal control) groups. OPLS‐DA models were validated by permutation tests. R2Y (the coefficient of determination for the Y matrix, representing the proportion of variance explained by the model) and Q2Y (the cross‐validated predictive coefficient for the Y matrix, reflecting the model’s predictive ability) were used to evaluate the goodness of fit and predictive ability of the models, respectively. The variable importance in projection (VIP) generated in OPLS‐DA processing represents the contribution to the discrimination of each metabolite ion between the groups. Metabolites with VIP > 2 and *p* < 0.05 were considered as significant differential metabolites. Random forest analysis was performed using the OmicStudio tools (https://www.omicstudio.cn/tool) to screen for key differential metabolites with high discriminative power. Functional enrichment analysis of the identified differential metabolites was conducted using the KEGG database to explore their involvement in biological pathways.

## 3 Results

### 3.1 General Description of Plasma Metabolomics Data

**FIGURE 1:**
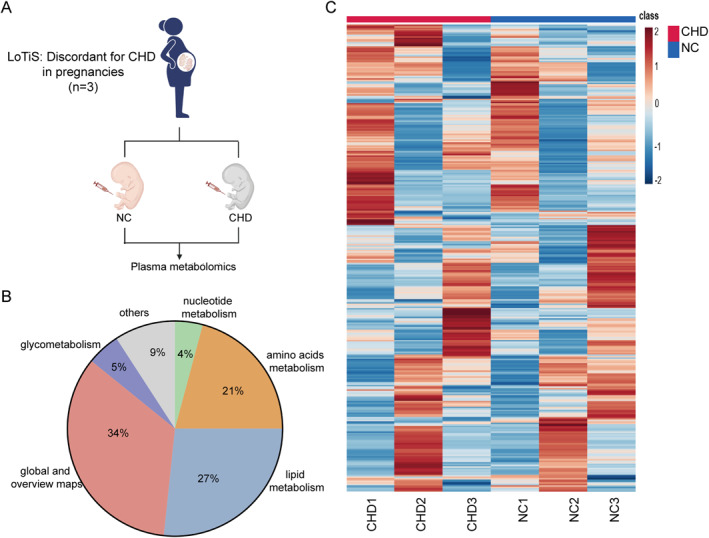
Overview of the study design and metabolic profiling of cord blood plasma in twin pregnancies discordant for CHD. (A) Schematic of study design and participants. Three twin pregnancies discordant for CHD were included: Each pregnancy contained one fetus with CHD and one normal control (NC) fetus. Cord blood plasma samples were collected from both fetuses per pair, and plasma metabolomic analysis was performed to characterize metabolic signatures. NC, normal control (fetus without CHD); CHD, congenital heart disease (affected fetus). (B) Pie chart displaying the functional classification of identified metabolites in cord blood plasma. Metabolites were grouped into 6 categories based on annotated biological functions: global and overview maps (34%), lipid metabolism (27%), amino acids metabolism (21%), others (9%), glycometabolism (5%), and nucleotide metabolism (4%). (C) Hierarchical clustering analysis heat map of metabolic profiles between the CHD and NC groups. Due to the large number of metabolites analyzed, individual metabolite names are not labeled on the y‐axis to maintain visual clarity. The color scale and clustering pattern are the primary focus of this figure, highlighting the distinct metabolic profiles between groups. Color intensity (red to blue) represents standardized relative metabolite abundance: Red indicates higher levels, and blue indicates lower levels.

We used a nontargeted metabolomics approach to analyze plasma from three pairs of CHD‐discordant MZ twins (Figure 1A). A total of 323 metabolites in umbilical cord blood were quantified based on compound libraries. Among them, 120 metabolites were involved in different pathways. The highest percentage of these metabolites was lipid‐related (27%) and amino acid–related (21%) (Figure 1B). Using an unsupervised hierarchical clustering method to further investigate the difference in plasma metabolome between CHD and NC groups, the heat map revealed that cord blood plasma in twins with CHD had a different metabolic pattern from that of the NC group (Figure 1C).

### 3.2 OPLS‐DA and Random Forest Analyses of Metabolites in Umbilical Cord Plasma From CHD‐Discordant Twins

**FIGURE 2:**
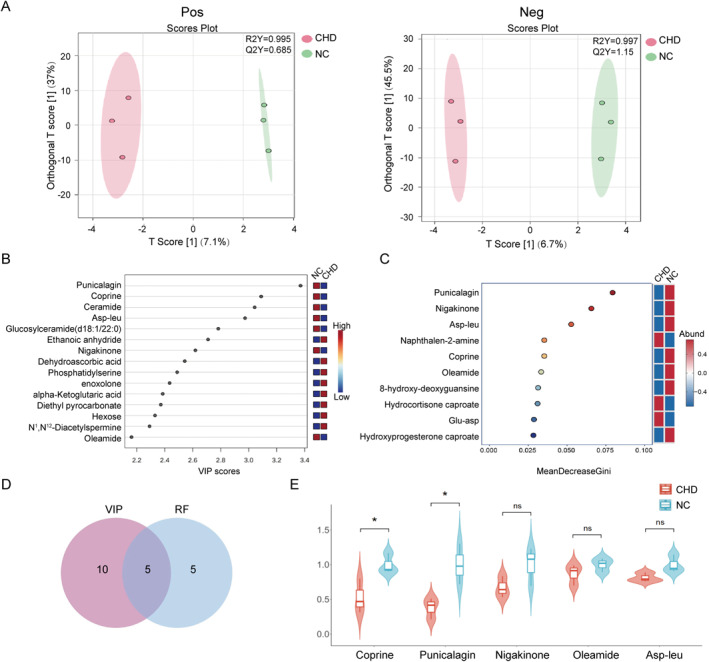
Separation of cord blood plasma metabolic profiles between the congenital heart disease (CHD) and normal control (NC) groups. (A) Orthogonal partial least squares discriminant analysis (OPLS‐DA) score plots comparing metabolic plasma of cord blood. Scores plot generated from the OPLS‐DA model shows CHD (red) as compared with the control (green) in positive ion mode (left, pos) and negative ion mode (right, neg). R2Y indicates the model's goodness of fit; Q2Y indicates the model's predictive ability. (B) Variable importance in projection (VIP) scores of the top 15 metabolites contributing to the separation between CHD and NC groups. The VIP score reflects the metabolite's contribution to OPLS‐DA discriminative ability. The color gradient (from blue to red) corresponds to the relative abundance of each metabolite in the CHD group. (C) Random forest (RF) analysis of metabolite importance for distinguishing CHD from NC. The color gradient (from blue to red) indicates the metabolite's relative abundance. (D) Venn diagram of biomarkers selected by both VIP and RF methods. (E) Normalized concentrations of the five overlapping biomarkers in CHD (red) and NC (blue) groups. ns, not significant; *p < 0.05.

We compared the different metabolites in positive and negative ion modes between the CHD and NC groups. OPLS‐DA, as a powerful statistical modeling tool, was used for predictions to identify differentially expressed metabolites between the CHD and NC groups. The results demonstrated that metabolites in the umbilical cord plasma samples between the two groups showed a clear separation (Figure 2A). The variance of the response variable (R2Y) in the positive and negative modes was 0.995 and 0.997, respectively. While visual separation was observed between the groups, the data were overfitted, which is likely attributable to the small sample size; the variance for modeling in cross‐validation (Q2Y) was −0.685 and −1.15, respectively. The VIP values of the metabolite were greater than 1.5. This indicates a significant contribution to the model (Table 1). The top 15 metabolites that contributed most to the differences between NC and CHD subjects are shown in Figure 2B.

**TABLE 1:** The OPLS‐DA parameters of differential expressed metabolites.

No.	Metabolite name	VIP	*p* [1]	*p* (corr)
1	Punicalagin	3.370616232	4.0895	0.87684
2	Coprine	3.08912942	3.7479	0.80362
3	C24:1 ceramide	3.042956622	3.6919	0.79161
4	Asp‐Leu	2.973524291	3.6077	0.77354
5	C22 glucosylceramide	2.783098709	3.3766	0.72401
6	Ethanoic anhydride	2.708873258	−3.2866	−0.7047
7	Nigakinone	2.619261409	3.1779	0.68138
8	DHA	2.543188441	−3.0856	−0.66159
9	Phosphatidylserine	2.488034451	−3.0187	−0.64725
10	Enoxolone	2.434738213	−2.954	−0.63338
11	Alpha‐KG	2.384977112	−2.8936	−0.62044
12	Diethylpyrocarbonate	2.371759426	−2.8776	−0.617
13	Hexose	2.329889047	−2.8268	−0.60611
14	DiAcSpm	2.29122878	−2.7799	−0.59605
15	Oleamide	2.16209932	2.6232	0.56246
16	N‐lauroylglycine	2.153271606	−2.6125	−0.56016
17	Asarone	2.122456629	−2.5751	−0.55214
18	Thiolutin	2.106220241	−2.5554	−0.54792
19	EDTA	2.100817814	2.5489	0.54651
20	G12	2.08713977	−2.5323	−0.54296
21	LPC	2.028510896	−2.4611	−0.5277
22	Harmaline	1.961805684	2.3802	0.51035
23	Piceatannol	1.895847075	−2.3002	−0.49319
24	Aspirin	1.881150536	−2.2823	−0.48937
25	Coenzyme Q2	1.854034901	2.2494	0.48232
26	(3E)‐4,8‐dimethyl‐1,3,7‐nonatriene	1.848294717	−2.2425	−0.48082
27	Carboxycyclophosphamide	1.843202012	2.2363	0.4795
28	LysoPC (22:1[13Z])	1.840485438	−2.233	−0.47879
29	Diquafosol	1.830217987	−2.2205	−0.47612
30	Hymecromone	1.822310221	−2.211	−0.47406
31	Boldione	1.786541866	−2.1676	−0.46476
32	Hexanoic acid	1.687028478	2.0468	0.43887
33	(2E)‐3‐(3,4‐dimethoxyphenyl) acrylic acid	1.686674941	−2.0464	−0.43878
34	3‐Furoic acid	1.654192446	−2.007	−0.43033
35	3, 5‐Tetradecadiencarnitine	1.64451785	−1.9952	−0.42781
36	ACPC	1.642658774	−1.993	−0.42733
37	17‐Hydroxypregnenolone sulfate	1.625753907	1.9725	0.42293
38	2,3,4,5‐Tetrahydroxypentanal	1.614065966	−1.9583	−0.41989
39	Emblicanin B	1.604760383	−1.947	−0.41747
40	12‐Hydroxylauric acid	1.604246302	−1.9464	−0.41733
41	N,N‐dimethylarginine	1.593590145	1.9335	0.41456
42	Methionine	1.586344638	1.9247	0.41268
43	Hydroxyprogesterone caproate	1.582638568	1.9202	0.41171
44	Mollicellin B	1.564293581	−1.8979	−0.40694
45	2,6‐di‐tert‐butylhydroquinone	1.561179445	1.8941	0.40613
46	DL‐2,6‐diaminopimelic acid	1.531170695	1.8577	0.39832
47	DIMBOA	1.513650066	1.8365	0.39377
48	LysoPC (22:4 [7Z,10Z,13Z,16Z])	1.506861339	1.8282	0.392

Abbreviations: alpha‐KG, alpha‐ketoglutaric acid; ACPC, 1‐Aminocyclopropanecarboxylic acid; DiAcSpm, N^1^,N^12^‐diacetylspermine; DHA, Docosahexaenoic acid; DIMBOA, 2,4‐dihydroxy‐7‐methoxy‐1,4‐benzoxazin‐3‐one; EDTA, Ethylenediaminetetraacetic acid; G12, 12‐Hydroxydodecanoic acid; LPC, 1‐stearoyl‐sn‐glycero‐3‐phospho‐1D‐myo‐inositol; OPLS‐DA, orthogonal partial least square‐discriminate analysis; VIP, variable importance in projection.

We then employed a random forest (RF) algorithm, ranking metabolite importance through mean decrease Gini evaluation, to select the top 10 significant biomarker candidates (Figure 2C). Inter‐method agreement between the machine learning approaches was assessed via Venn diagram analysis, demonstrating concordant identification of five common metabolites: punicalagin, coprine, Asp‐Leu, nigakinone, and oleamide (Figure 2D). The box plots further characterized these metabolites. Punicalagin and coprine showed significant differences with lower levels in CHD group, suggesting a potential association with CHD (Figure 2E).

### 3.3 Distinct Metabolic Profiles of CHD‐Discordant Twins

**FIGURE 3:**
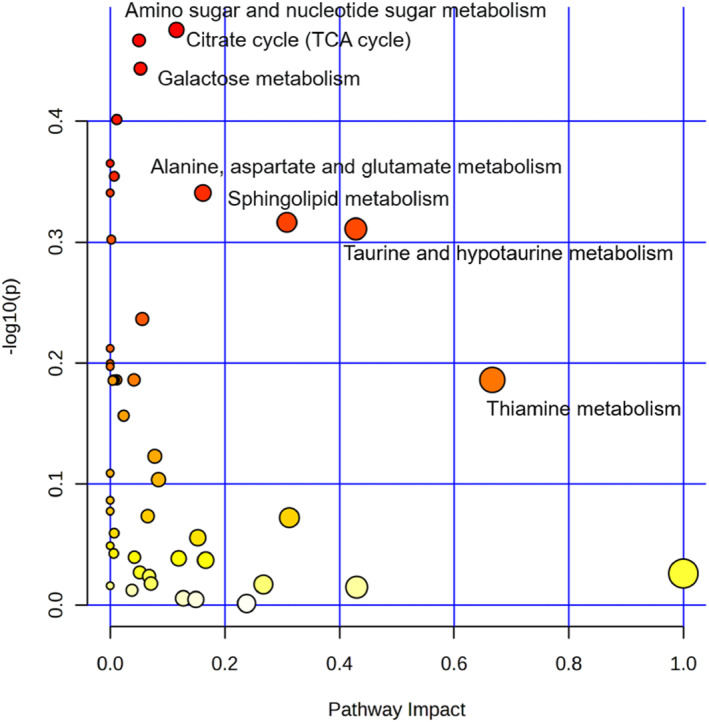
Topological pathway analysis of the umbilical cord plasma metabolites between congenital heart disease (CHD) and normal control (NC) groups. This plot presents the results of quantitative enrichment analysis for metabolic pathways linked to differential metabolites in umbilical cord plasma of CHD and NC groups. The horizontal axis reflects the topological importance of each pathway in the metabolic network (Higher values indicate greater structural impact). The vertical axis represents the enrichment significance of the pathway. The color of each sphere denotes enrichment significance (red = most significant and yellow = least significant). Labeled pathways (e.g., citrate cycle [TCA cycle] and galactose metabolism) are the most significantly altered metabolic pathways between the two groups.

The metabolic pathway analysis was enriched with annotated metabolites from the umbilical cord plasma, which distinguished the CHD and NC groups. The most highly enriched and relevant metabolic pathways included thiamine metabolism, taurine and hypotaurine metabolism, sphingolipid metabolism, alanine, aspartate and glutamate metabolism, the citrate (TCA) cycle, galactose metabolism, and amino sugar and nucleotide sugar metabolism (Figure 3).

### 3.4 The Difference in Metabolites Expressed Between CHD and NC Groups

**FIGURE 4:**
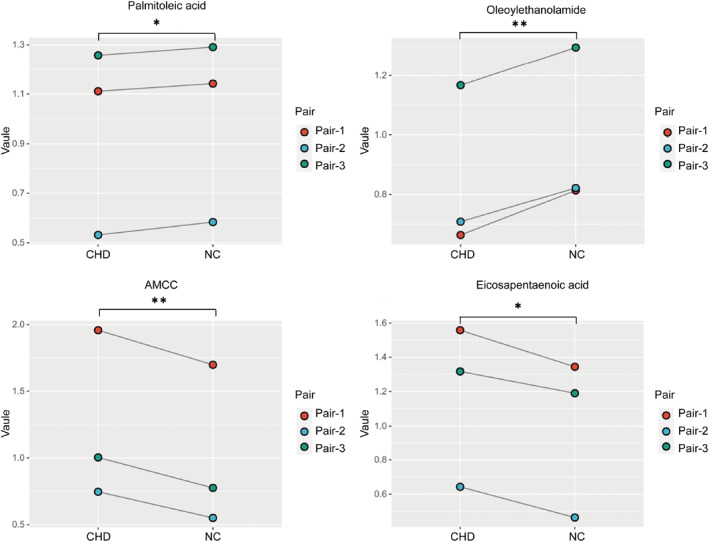
Four typical differentially expressed metabolites (DEMs) between congenital heart disease (CHD) and normal control (NC) groups. This figure displays the normalized plasma concentrations of four typical DEMs in umbilical cord blood samples (from CHD and NC groups) derived from 3 discordant twin pairs (Pair 1, Pair 2, and Pair 3; distinguished by different colors). Data are analyzed using a paired‐samples *t*‐test. *p < 0.05, **p < 0.01.

MZ pairs share 100% of the gene sequences, and discordant twin pairs are informative as regarding the contribution of environmental effects [22]. Hence, we applied two‐sided paired‐sample *t*‐test to analyze differentially expressed metabolites (DEMs) between CHD and NC groups. Typical DEMs are shown in Figure 4: palmitoleic acid (POA) and oleoylethanolamide (OEA) were significantly decreased in the CHD group. Furthermore, N‑Acetyl‑S‑(N‑methylcarbamoyl)‑L‑cysteine (AMCC) and eicosapentaenoic acid (EPA) were significantly increased in the CHD group.

## 4 Discussion

Many studies have demonstrated that the changes in specific metabolites are closely related to CHD pathogenesis. It has also been found that plasma metabolomics analysis is of great value in revealing the molecular mechanisms of CHD and identifying biomarkers for diagnosis and treatment [23, 24]. In this study, we first selected cord blood from MZ twins discordant for CHD for a metabolomics study based on UHPLC‐MS/MS to describe the different metabolic profiles.

The OPLS‐DA model showed that CHD and NC groups had distinct plasma metabolic profiles in cord blood; however, we could not reliably distinguish between CHD and NC subjects based solely on global metabolic profiles. Failure to achieve statistical separation is very likely due to the smaller number of cases than the number of metabolites being analyzed in the study, a key limitation inherent to this exploratory investigation. Therefore, combined with the RF analysis method, we screened out the possible common important metabolites: punicalagin and coprine. Additionally, the results of pathway enrichment analysis showed disease‐specific metabolic signatures and indicated that amino acid and nucleotide metabolism, lipid metabolism, and energy metabolism are involved in CHD. These findings are preliminary and require validation in larger, independent cohorts.

Notably, the concurrent disruption of amino acid, lipid, and glucose metabolism pathways observed in our study suggests a potential anomaly in a common upstream regulatory hub. The mammalian target of rapamycin (mTOR) signaling pathway serves as a central integrator of nutrient sensing and metabolic regulation, directly responding to amino acid‐, glucose‐, and lipid‐derived signals while playing a pivotal role in cardiovascular development [25, 26]. For instance, amino acids (a key class of differentially abundant metabolites in our study) can activate mammalian target of rapamycin complex 1 (mTORC1) to modulate downstream anabolic processes and metabolic homeostasis; meanwhile, nuclear mTOR signaling orchestrates transcriptional programs underlying cellular growth and metabolic adaptation, which are critical for normal cardiac morphogenesis [27]. Based on these insights, we speculate that the metabolomic differences identified in cord blood may reflect adaptive regulation of the mTOR signaling pathway in response to intrauterine environmental changes (e.g., nutritional fluctuations or stress). This association provides an integrative mechanistic framework for understanding how multiple metabolic disturbances could jointly contribute to abnormal cardiac development, linking our observed metabolic signatures to a well‐characterized pathway involved in CHD pathogenesis.

In univariate analysis, only changes in punicalagin and coprine were verified and the concentrations of two metabolites were significantly decreased in CHD. Punicalagin, the primary ellagitannin polyphenol found in the peel or seeds of the pomegranates, raspberries, strawberries, and walnuts, has been demonstrated to have antioxidant and anti‐inflammatory properties in cardiovascular diseases [28]. As a potential safe and effective therapeutic agent for cardiovascular disorders, punicalagin has good myocardial protection, which significantly enhances cardiac function and decreases the size of infarcts [29, 30]. However, the underlying mechanism linking reduced punicalagin levels to CHD remains to be elucidated. While variations in maternal dietary intake represent a plausible explanation, other factors such as differential placental transfer efficiency or fetal metabolic reprogramming in response to the disease state could equally account for the observed association. Coprine is a precursor of 1‐aminocyclopropanol, a toxic substance which blocks the normal breakdown of alcohol to acetic acid by inhibiting the enzyme aldehyde dehydrogenase [31]. No studies have yet reported its association with CHD, and more in‐depth studies are needed in the future.

Interestingly, with paired‐sample *t*‐test in MZ twins discordant for CHD, four significant DEMs emerged. Of these, the concentrations of three fatty acids including POA, OEA, and EPA were dysregulated in CHD. However, Chen et al. [32] found a significant increase in POA levels in peripheral blood of patients with pulmonary arterial hypertension. In contrast to our results, these discrepancies may be caused by the selected sample type because our study of the plasma of cord blood is more indicative of maternal risk during pregnancy. OEA is a bioactive endogenous monounsaturated lipid molecule synthesized by oleic acid [33], whereas POA, an *ω*‐7 monounsaturated fatty acid (MUFA) and EPA, a long‐chain *ω*‐3 polyunsaturated fatty acid (PUFA) could be derived from fatty fish, fish oil, and some nuts [34]. Numerous studies have examined evidence supporting that foods containing unsaturated fatty acids could reduce the risk of developing cardiovascular disease [35, 36, 37]. In the present study, we found an imbalance of unsaturated fatty acids in cord blood plasma of CHD. This observed dysregulation may be linked to several prenatal factors, including maternal dietary patterns, differential placental transfer of specific fatty acids, or fetal metabolic adaptations. Collectively, these factors could shape an altered intrauterine metabolic milieu, potentially associated with the observed alterations in metabolites.

In this study, we comprehensively performed quantitative metabolomic techniques to reflect the relationship of cord blood plasma metabolites of MZ twins discordant for CHD. Our analyses suggest that the intrauterine metabolic environment, as reflected in cord blood, may be associated with fetal cardiovascular development. This perspective could inform future strategies of antenatal monitoring and the exploration of preventive approaches for CHD. It is important to note the exploratory nature of this investigation, which is primarily due to the limited sample size. Within the larger LoTiS cohort of 439 twin pregnancies, only three CHD‐discordant MZ twin pairs were identified. This small sample size constrains the statistical power and limits the generalizability of our findings. Therefore, the specific metabolites and pathways identified should be considered as generating valuable hypotheses rather than establishing definitive biomarkers. Nevertheless, this study highlights the unique utility of the discordant MZ twin model combined with cord blood metabolomics by minimizing confounding factors from genetic background and shared maternal factors. It provides a direct window into the prenatal metabolic milieu distinct from maternal peripheral blood, offering novel and specific clues about the early life origins of CHD. To translate these promising leads into clinical insights, future research with larger, independent cohorts is essential to validate our findings and elucidate the underlying biological mechanisms driving the observed metabolic alterations.

## Author Contributions

Fang Xiang analyzed the data and drafted the manuscript. Xiao Yuan aided in sample collection and data pre‐processing. Yi Yang contributed to metabolomic data analysis and interpretation. Philip N. Baker, Mark D. Kilby, and Chao Tong contributed to the collection of the clinical cohort LoTiS and provided related research guidance, and Chao Tong also revised the manuscript. Xi Yuan conceived the study, supervised the research, and finalized the manuscript.

## Ethics Statement

The study protocol was approved by the Ethics Committee of the First Affiliated Hospital of Chongqing Medical University (No. 2021–646).

## Conflicts of Interest

The authors declare no conflicts of interest.

## Data Availability

The raw data that support the findings of this study are available from the corresponding author upon reasonable request.
